# Effects of Plant Extracts on Human Health—2nd Edition

**DOI:** 10.3390/nu18050732

**Published:** 2026-02-25

**Authors:** Fátima Regina Mena Barreto Silva, Marcela Aragón Novoa

**Affiliations:** 1 Instituto de Bioeletricidade Celular (IBIOCEL): Ciência & Saúde, Departamento de Bioquímica, Universidade Federal de Santa Catarina, Florianópolis 88049-900, SC, Brazil; 2 Departamento de Farmacia, Universidad Nacional de Colombia, Av. Carrera 30 # 45-03 Edif 450, Bogotá 111321, Colombia

Plant extracts and their bioactive components have garnered increasing scientific interest due to their diverse chemical profiles and potential impacts on human health. From antioxidant and anti-inflammatory activity to metabolic and neuroprotective effects, botanicals continue to be explored across nutritional, physiological, and clinical domains. The Special Issue “Effects of Plant Extracts on Human Health—2nd Edition” in *Nutrients* assembles a multidisciplinary collection of articles that collectively advance our understanding of these complex interactions and highlight emerging directions in plant extract research.

A core theme that emerges from this Special Issue is the investigation of plant extracts in the context of metabolic health. Several contributions examine how specific botanical extracts influence lipid metabolism, glycemic control, and weight management, offering insights into mechanisms that may complement traditional dietary and pharmacological interventions. These studies underscore the importance of standardized extract characterization and appropriate dosimetry in assessing physiological outcomes, and they point toward the need for rigorous human trials to substantiate preclinical findings.

Another significant strand of research in this Special Issue focuses on the neuroprotective potential of plant-derived compounds. Evidence from both cellular and animal models suggests that specific phytochemicals can modulate signaling pathways associated with neuronal survival, oxidative stress, and inflammation. These findings contribute to a growing body of literature that positions certain plant extracts as candidates for mitigating age-related cognitive decline and supporting neural health, although translation to clinical contexts remains a challenge.

The Special Issue also includes contributions that explore the bioactive diversity of lesser-studied plant species and their unique phytochemical profiles. Such work expands the botanical landscape beyond commonly studied extracts, illuminating novel chemical entities and complex mixtures with promising bioactivity. This trend aligns with the broader objective of diversifying the sources and structural motifs under investigation, and it highlights the value of integrating advanced analytical technologies to map extract composition with biological function.

Across multiple contributions, several articles identify challenges associated with variable extract composition and inconsistent reporting practices, reinforcing calls for harmonized analytical protocols. These methodological considerations are essential for making meaningful comparisons across studies and for advancing plant extract research toward effective human applications.

Despite notable progress, several knowledge gaps remain. A limited number of well-powered clinical trials means that evidence for many putative health effects is still preliminary. Furthermore, uncertainties about optimal dosing regimens, long-term safety, and interactions with conventional therapies highlight the need for careful translational research. The complexity of plant extracts—often comprising hundreds of compounds with potential synergistic interactions—adds an additional layer of challenge that warrants integrative biological and computational approaches.

Looking forward, the collection of work published in this Special Issue suggests several promising directions for future research. Studies that bridge pharmacokinetics, systems biology, metabolomics, and clinical nutrition are likely to yield deeper mechanistic insights and facilitate personalized approaches to botanical interventions. Additionally, an expanded focus on bioavailability enhancement strategies and targeted delivery systems may improve the efficacy and consistency of plant-derived bioactives in human populations.

“Effects of Plant Extracts on Human Health—2nd Edition” provides a timely overview of contemporary research exploring the multifaceted roles of plant extracts in human health. The breadth of topics covered—ranging from metabolic modulation to neuroprotection and extract characterization—reflects the dynamic nature of the field and underscores the potential of plant-based approaches to contribute to nutritional and therapeutic strategies. We hope that this Special Issue will stimulate further research, foster interdisciplinary collaboration, and support the responsible translation of botanical insights into health-promoting applications.

